# Lymphatic‐specific methyltransferase‐like 3‐mediated m^6^A modification drives vascular patterning through prostaglandin metabolism reprogramming

**DOI:** 10.1002/mco2.728

**Published:** 2024-10-04

**Authors:** Lianjun Shi, Shuting Lu, Xue Han, Fan Ye, Xiumiao Li, Ziran Zhang, Qin Jiang, Biao Yan

**Affiliations:** ^1^ Department of Ophthalmology and Optometry The Affiliated Eye Hospital, Nanjing Medical University China; ^2^ The Fourth School of Clinical Medicine Nanjing Medical University Nanjing China; ^3^ Department of Ophthalmology Shanghai General Hospital Shanghai Jiao Tong University School of Medicine Shanghai China

**Keywords:** corneal neovascularization, lymphangiogenesis, m^6^A modification, METTL3, prostaglandin metabolism

## Abstract

Lymphangiogenesis plays a pivotal role in the pathogenesis of various vascular disorders, including ocular vascular diseases and cancers. Deregulation of *N* ^6^‐methyladenosine (m^6^A) modification has been identified as a key contributor to human diseases. However, the specific involvement of m^6^A modification in lymphatic remodeling remains poorly understood. In this study, we demonstrate that inflammatory stimulation and corneal sutures induce elevated levels of methyltransferase‐like 3 (METTL3)‐mediated m^6^A modification. METTL3 knockdown inhibits lymphatic endothelial viability, proliferation, migration, and tube formation in vitro. METTL3 knockdown attenuates corneal sutures‐induced lymphangiogenesis and intratumoral lymphangiogenesis initiated by subcutaneous grafts, consequently restraining corneal neovascularization, tumor growth, and tumor neovascularization in vivo. Mechanistically, METTL3 knockdown upregulates prostaglandin–endoperoxide synthase 2 expression through an m^6^A–YTHDF2‐dependent pathway, enhancing the synthesis of cyclopentenone prostaglandins (CyPGs). Aberrant CyPG production in lymphatic endothelial cells impairs mitochondrial oxidative phosphorylation, contributing to pathological lymphangiogenesis. Moreover, selective inhibition of METTL3 with STM2457 reduces m^6^A levels in lymphatic endothelial cells, effectively suppressing pathological lymphangiogenesis. This study provides compelling evidence that lymphatic‐specific METTL3 plays a critical role in vascular patterning through prostaglandin metabolism reprogramming. Thus, METTL3 emerges as a promising target for treating lymphangiogenesis‐related diseases.

## Graphical abstract

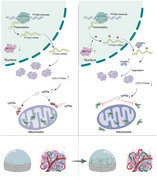

## 1 INTRODUCTION

The lymphatic system and vascular system are two important systems in the human body. The lymphatic system is characterized as a linear, blind‐ended system comprising lymphatic vessels and lymphoid organs.^1^ Under physiological conditions, lymphatic system plays crucial roles in several biological processes, such as maintaining fluid homeostasis, absorbing lipids and vitamins, and supporting host immune defense.^2^, ^3^ However, under diseased conditions, lymphatic endothelial cells become activated and exhibit abnormal proliferation, migration, and tube formation. Abnormal proliferation of lymphatic vessels, termed lymphangiogenesis, can disrupt fluid homeostasis, compromise immune function, and lead to the accumulation of lipid‐rich lymphatic fluid.^4^ Previous studies have revealed the involvement of pathological lymphangiogenesis in various diseases, including lymphedema, inflammation, transplant rejection, tumor metastasis, neurodegeneration, and cardiovascular disease.^5^, ^6^, ^7^ Thus, targeting lymphangiogenesis is a promising strategy for treating these diseases.

Lymphangiogenesis is a complex process driven by both genetic and epigenetic factors. Epigenetic mechanisms, including DNA methylation, chromatin remodeling, histone modifications, and RNA methylation, contribute to the development of this pathological process.^4^, ^8^, ^9^ Numerous posttranscriptional modifications occur at the RNA level, with *N* ^6^‐methyladenosine (m^6^A) methylation emerging as the most abundant modification in cellular RNAs.^10^ m^6^A methylation can regulate splicing, stability, and translation of mRNAs, thus affecting the levels of the corresponding proteins.^11^ m^6^A modification is known as a critical regulator of many processes, such as stem cell differentiation, nervous system development, and circadian rhythm. Abnormal m^6^A modification has been detected in several human diseases, including cancers, metabolic diseases, and neurological diseases.^12^, ^13^, ^14^ However, the role of m^6^A methylation in lymphangiogenesis remains elusive.

The lymphatic systems and blood vessels are functionally interconnected. They function synergistically to preserve fluid and protein equilibrium, facilitate cellular nourishment, and sustain immunological processes. During tumorigenesis, cancer cells exploit the lymphatic and blood vessels to metastasize to distant sites. In inflammatory conditions, the lymphatic and vascular systems facilitate the transport of immune cells and antigens, significantly affecting inflammatory processes.^1^, ^15^, ^16^ Combined therapeutic strategies targeting both angiogenesis and lymphangiogenesis have demonstrated potential benefits for patients with cancer or neovascular diseases.^1^, ^17^

The cornea, lacking lymphatic vessels, offers a unique model for studying lymphangiogenesis. Tumors induce lymphatic vessel growth for metastasis, making xenograft models valuable for studying lymphangiogenesis.^18^ This study investigated the role of m^6^A modification in lymphangiogenesis using corneal suture model and xenograft model. Our findings reveal an obvious increase in the levels of m^6^A modification during lymphangiogenesis. Notably, METTL3 knockdown could effectively suppress pathological lymphangiogenesis through reprogramming of prostaglandin (PG) metabolism. These results highlight METTL3 as a promising therapeutic target for diseases characterized by pathological lymphangiogenesis.

## 2 RESULTS

### 2.1 Lymphangiogenesis is associated with METTL3‐mediated m^6^A modification

**FIGURE 1:**
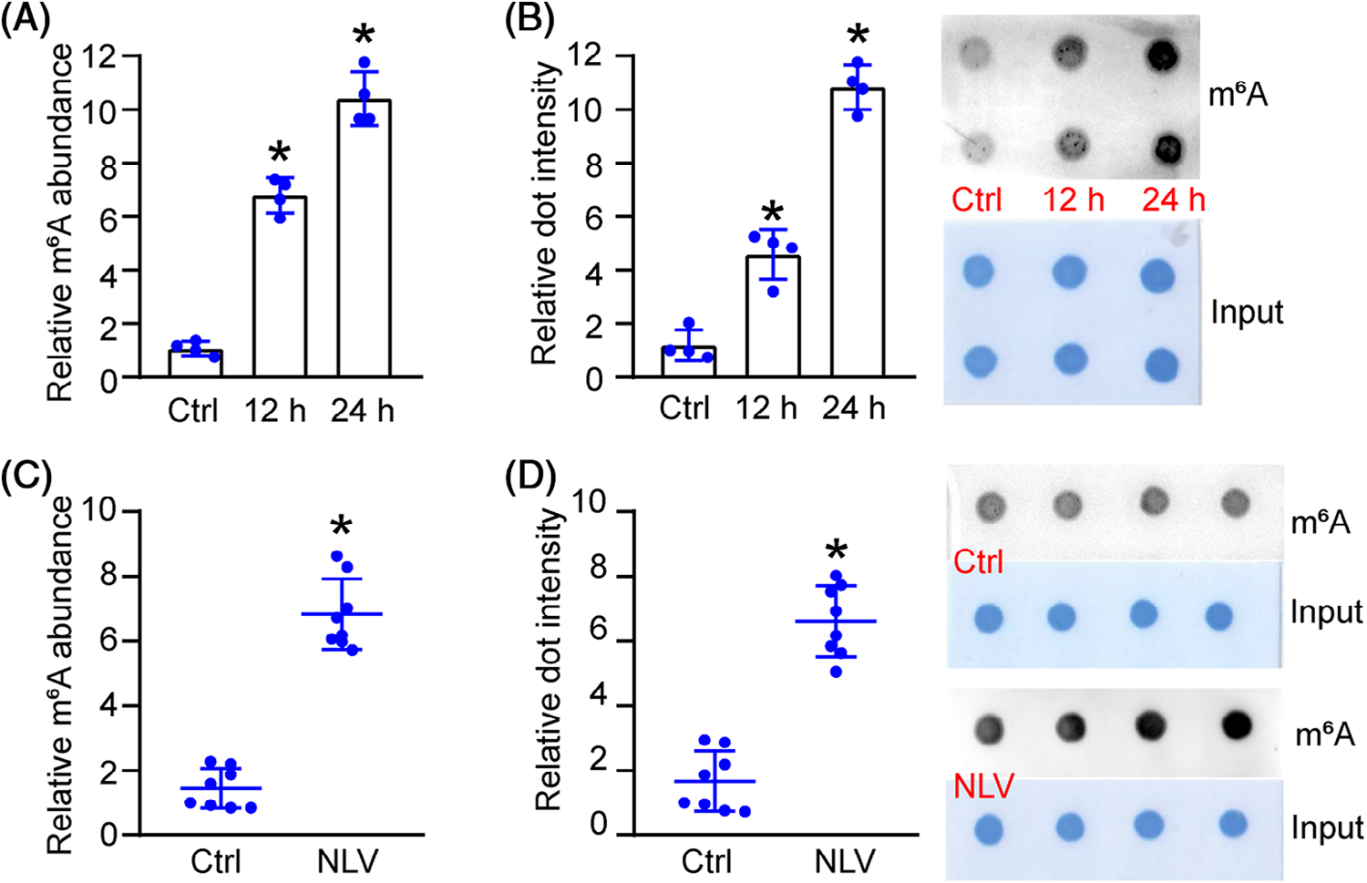
Lymphangiogenesis is associated with METTL3‐mediated m6A modification. (A and B) HLECs were incubated with LPS (1 μg/mL) to mimic inflammatory stress for 12 or 24 h. The group without LPS treatment was taken as the control (Ctrl) group. The levels of m6A RNA modification were detected by colorimetric quantification (A, *n* = 4, one‐way ANOVA with Bonferroni test) and dot blot assays (B, *n* = 4, one‐way ANOVA with Bonferroni test). (C and D) The levels of m6A RNA modification in the sutured corneas (NLV, new lymphatic vessels) and corresponding controls were determined by colorimetric quantification (C, *n* = 8 corneas per group, Mann–Whitney *U*‐test with Bonferroni test) and dot blot assays (D, *n* = 8 corneas per group, Mann–Whitney *U*‐test with Bonferroni test). *p < 0.05 versus Ctrl.

Lymphatic system is tightly associated with the homeostasis of tissue fluid and the trafficking of immune cells. Lymphangiogenesis occurs in vascular and nodal parts during inflammation and other diseased condition.^19^ Since inflammatory factors are known as the critical drivers for lymphangiogenesis, human lymphatic endothelial cells (HLECs) were exposed to lipopolysaccharide (LPS) to mimic pathological lymphangiogenesis in vitro. Total RNA was extracted and subjected to colorimetric quantification and dot blot assays. LPS exposure led to increased levels of m^6^A modification in HLECs (Figure 1A,B). The murine sutured cornea is a well‐established model for studying pathological lymphangiogenesis. Three 10‐0 corneal sutures were sewn equidistantly to the corneas. The corneas were harvested on day 7 postsuturing for RNA extraction. Both colorimetric quantification and dot blot assays demonstrated significantly elevated m^6^A modification levels in the sutured corneas compared with the controls (Figure 1C,D). Taken together, these results reveal a strong correlation between lymphangiogenesis and heightened m^6^A modification levels.

To elucidate the principal regulators implicated in the upregulation of m^6^A modification during lymphangiogenesis, HLECs were treated with LPS to induce pathological lymphangiogenesis. Subsequently, we assessed the expression profiles of m^6^A writers (METTL3, METTL14, and WTAP) and m^6^A erasers (FTO and ALKBH5). qRT‐PCR assays and western blots reveal a significant upregulation of METTL3 expression in response to LPS treatment relative to the control group (Figure S1A,B).

To substantiate the role of METTL3 in mediating increased m^6^A levels during lymphangiogenesis, we examined METTL3 expression levels in clinical corneal samples and sutured cornea model. The injured corneal fragments from the patients undergoing corneal transplantation and the corresponding donors were collected. qRT‐PCR analysis reveals a marked upregulation of METTL3 expression in recipient corneal fragments compared with donor samples (Figure S1C). Furthermore, Mettl3 expression levels were examined in the sutured mouse corneas, revealing a significant upregulation compared with normal corneas (Figure S1D,E). Collectively, these results suggest that lymphangiogenesis is associated with increased METTL3 expression both in vitro and in vivo.

### 2.2 METTL3 regulates lymphatic endothelial function in vitro

**FIGURE 2:**
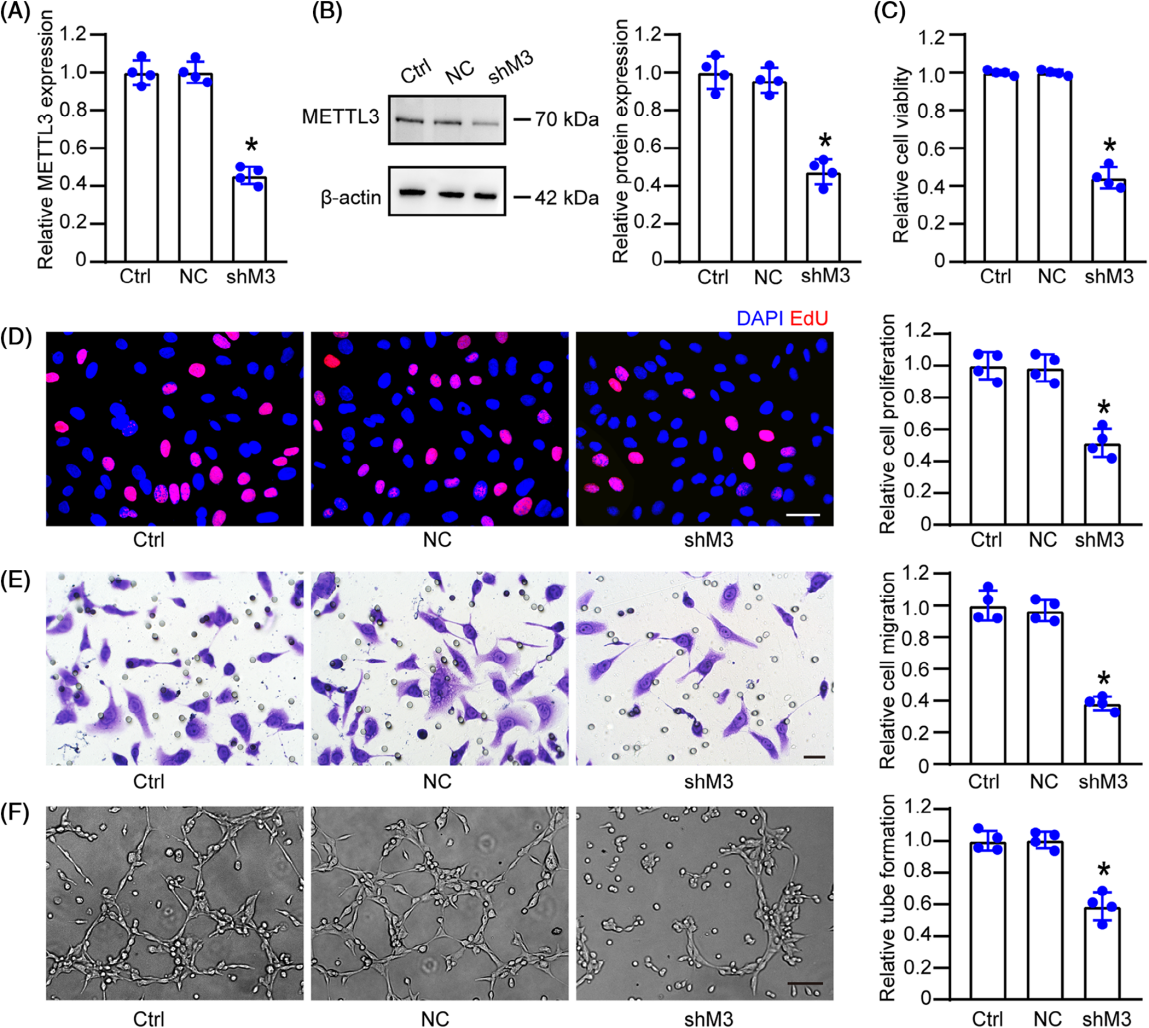
METTL3 knockdown inhibits lymphatic endothelial function in vitro. (A and B) HLECs were transfected with negative control shRNA (shC), METTL3 shRNA (shM3), or left untreated (Ctrl) for 24 h. qRT‐PCR assays were conducted to detect the levels of METTL3 mRNA (A, *n* = 4). Western blots were conducted to detect METTL3 protein levels, with β‐actin serving as the loading control (B, *n* = 4). MTT assays were conducted to detect cell viability (C, *n* = 4). Cell proliferation was assessed using the EdU detection kit, with quantification analysis provided. Blue, DAPI; red, EdU. Scale bar, 20 µm (D, *n* = 4). Transwell assay and quantification analysis was conducted to detect cell migration. Scale bar, 20 µm (E, *n* = 4). HLECs were seeded onto the Matrigel matrix and the tube‐like structures were observed at 6 h following cell seeding. Average tube length for each field was statistically analyzed. Scale bar, 50 µm (F, *n* = 4). Statistical significance was determined using one‐way ANOVA followed by Bonferroni's posthoc test, with *p < 0.05 versus Ctrl.

To determine whether METTL3 regulated lymphatic endothelial function in vitro, we engineered METTL3 short hairpin RNA (shRNA) to establish METTL3 knockdown HLECs or upregulated METTL3 levels through METTL3 overexpression. Compared with the control group, transfection of METTL3 shRNA significantly reduced the levels of METTL3 expression (Figure 2A,B), while METTL3 overexpression led to enhanced METTL3 levels in HLECs, which was similar to METTL3 expression in HLECs followed by inflammatory stress (Figure S2). Furthermore, METTL3 knockdown significantly decreased m^6^A levels, while METTL3 overexpression increased m^6^A levels under both normal and inflammatory conditions (Figure S3), suggesting that METTL3 is a crucial regulator of m^6^A modification in HLECs.

We then determined the significance of METTL3 in lymphatic endothelial function in vitro. MTT [3‐(4,5)‐dimethylthiahiazo (‐z‐y1)‐3,5‐di‐ phenytetrazoliumromide] assays demonstrated that transfection of METTL3 shRNA led to a marked reduction of HLEC viability (Figure 2C). EdU staining assays indicated that METTL3 knockdown led to an obvious reduction of HLEC proliferation (Figure 2D). Transwell assays and tube formation assays showed that transfection of METTL3 shRNA led to decreased migration and tube formation ability in HLECs (Figure 2E,F). We also determined whether METTL3 was sufficiently alone to drive lymphatic endothelial phenotype. METTL3 overexpression enhanced the viability, proliferation, migration, and tube formation ability of HLECs (Figure S4). Collectively, these findings demonstrate the critical role of METTL3 in modulating key aspects of lymphatic endothelial function in vitro.

**FIGURE 3:**
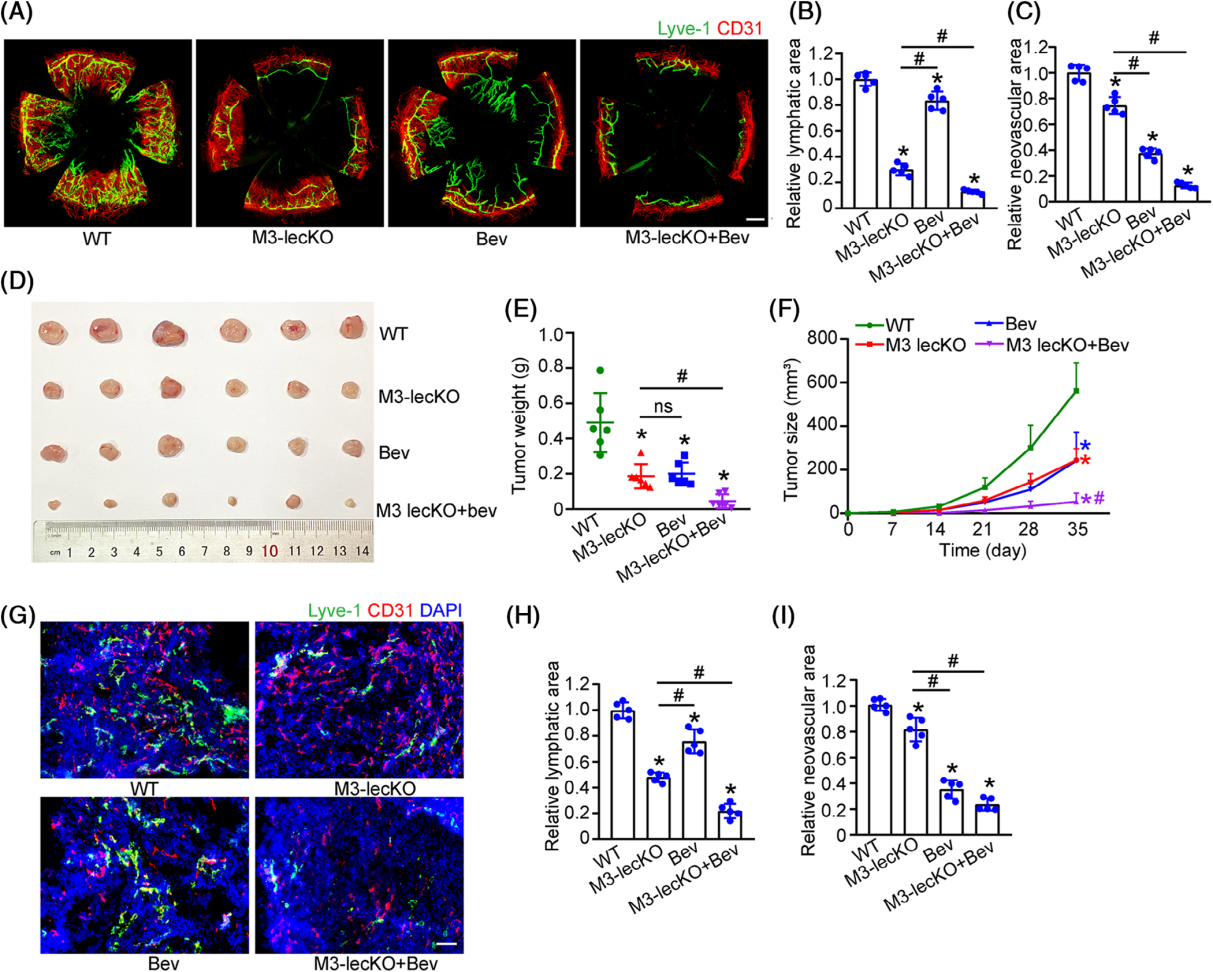
Lymphatic‐specific Mettl3 knockout suppresses pathological lymphangiogenesis and neovascularization. (A–C) Wide‐type mice and Mettl3–lecKO (M3–lecKO) mice were sutured with 10‐0 nylon sutures. Then, these mice were administrated with or without bevacizumab (Bev; 10 mg/mL) for 7 days. Finally, the corneas were harvested, flat‐mounted, and stained with CD31 (red) and LYVE‐1 (green) to label corneal lymphangiogenesis and neovascularization. Scale bar, 200 µm. Statistical analysis was conducted to compare the differences of lymphangiogenesis and neovascularization areas (*n* = 5, *p < 0.05 vs. Ctrl, #*p* < 0.05 vs. M3–lecKO, Mann–Whitney *U*‐test with Bonferroni test). (D–F) Wide‐type and M3–lecKO mice were injected subcutaneously with U87 cells plus moderate Matrigel on one side of lower abdomen, followed by administration with or without bevacizumab around subcutaneous masses for the indicated time points. After 35 days, tumor masses were removed, photographed, and weighed. Tumor weight was statistically analyzed and the weight of nude mice was recorded (*n* = 6, *p < 0.05 vs. Ctrl, #*p* < 0.05 vs. M3–lecKO, one‐way ANOVA with Bonferroni test). (G–I) Tumor masses were cut into 10‐µm thickness sections. Then, they were stained with CD31 (red) and LYVE‐1 (green) to label corneal lymphangiogenesis and neovascularization. DAPI was stained to label nuclei. Scale bar, 100 µm (*n* = 5, *p < 0.05 vs. Ctrl, #*p* < 0.05 vs. M3–lecKO, Mann–Whitney *U*‐test with Bonferroni test).

### 2.3 Lymphatic‐specific Mettl3 knockout suppresses pathological lymphangiogenesis and neovascularization

We then determined the role of lymphatic‐specific Mettl3 in lymphangiogenesis using a corneal sutured model. Breeding between heterozygous Mettl3^flox/+^ mice produced offspring following a Mendelian ratio, resulting in homozygous Mettl3^flox/flox^ mice. These Mettl3^flox/flox^ mice were viable, fertile, and exhibited no sarcomeric or histological abnormalities. Mettl3^flox/flox^ mice were crossed with Lyve1–IRES–iCre mice, which are activated by tamoxifen, to generate lymphatic‐specific Mettl3 knockout mice (Mettl3–lecKO). The levels of Mettl3 expression were significantly lower in the corneas of Mettl3–lecKO mice than that in wild‐type mice (Figure S5A,B). CD31/LYVE‐1 double staining was performed to detect the regions of corneal neovascularization and lymphangiogenesis. Compared with wild‐type group, Mettl3–lecKO mice had reduced lymphangiogenic areas in corneal sutured model. Administration of bevacizumab had a slight inhibitory effect on lymphangiogenesis. Notably, Mettl3–lecKO plus bevacizumab had the best inhibitory effects on lymphangiogenesis. As for corneal neovascularization, both bevacizumab administration and Mettl3–lecKO could reduce the angiogenic area in a corneal sutured model. Bevacizumab was more effective than Mettl3–lecKO in suppressing corneal neovascularization. Furthermore, Mettl3–lecKO plus bevacizumab had the best antianiogenic effects in the corneal sutured model (Figure 3A–C). We also detected the levels of m^6^A modification in each group. The results showed that Mettl3–lecKO reduced m^6^A abundance, while bevacizumab treatment had no effect on the abundance of m^6^A level (Figure S5C).

The impact of Mettl3–lecKO on lymphangiogenesis and its potential synergy with neovascularization was assessed using a tumor model. U87 MG glioblastoma cells were subcutaneously implanted into wild‐type and Mettl3–lecKO mice. Bevacizumab was administered weekly peritumorally. As expected, either Mettl3–lecKO or bevacizumab administration inhibited tumor growth. Mettl3–lecKO plus bevacizumab administration achieved the optimal antitumor effects but did not affect the whole body weight (Figures 3D–F and S5D). Tissue slicing also verified that Mettl3–lecKO significantly inhibited lymphangiogenesis in tumor masses. The combination of Mettl3–lecKO and bevacizumab exhibited optimal therapeutic efficacy against pathological lymphangiogenesis and neovascularization (Figure 3G–I).

### 2.4 METTL3 regulates PG metabolism in HLECs

**FIGURE 4:**
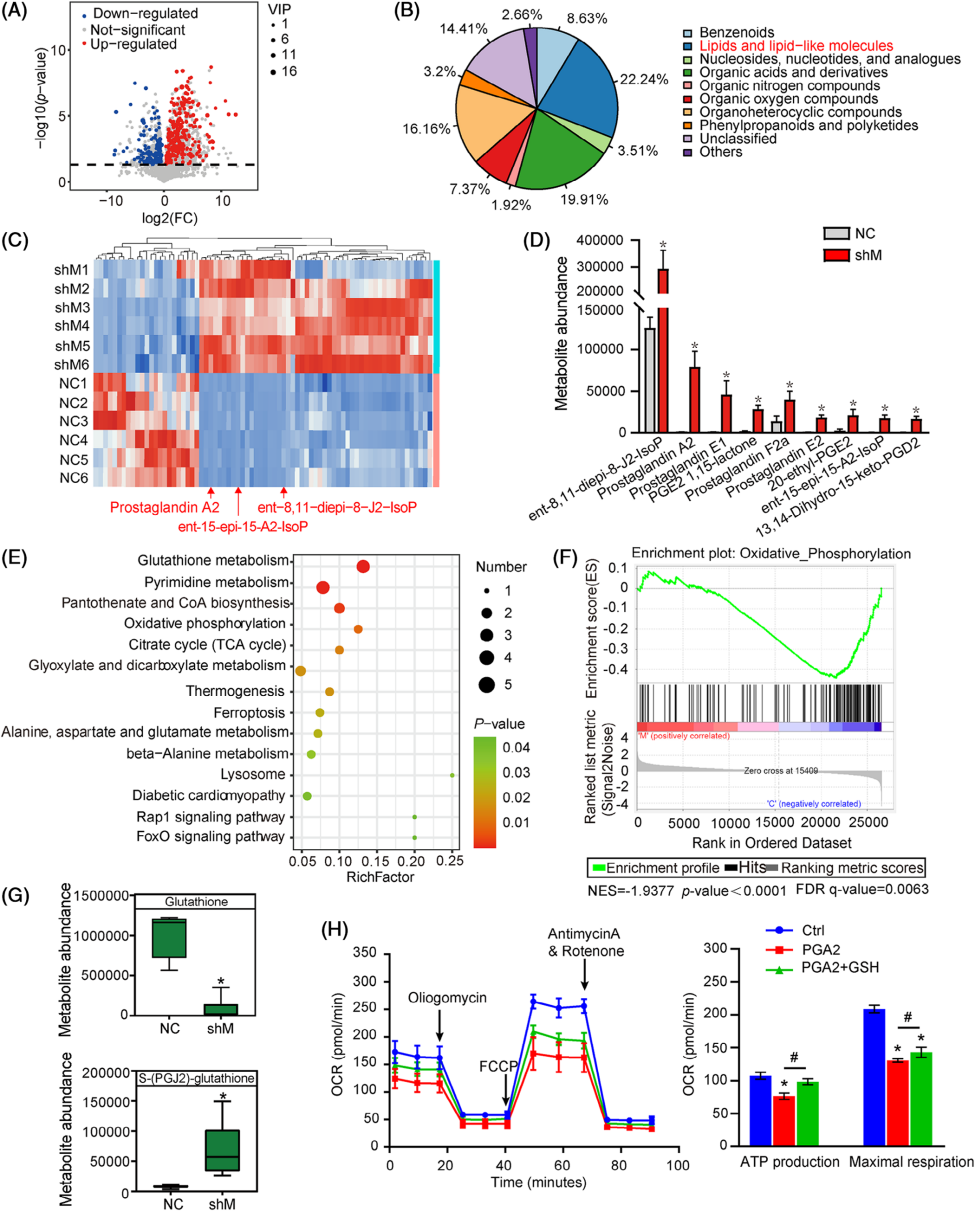
METTL3 regulates prostaglandin metabolism in HLECs. (A) Volcano plot filtering of differential metabolites between HLECs transfected with METTL3 shRNA or negative control (NC) shRNA by univariate analysis. Red dots: upregulated metabolites (VIP, variable importance in projection; *p *< 0.05, VIP > 1, and FC > 1); Blue dots, downregulated metabolites (*p *< 0.05, VIP > 1, and FC > 1, *n* = 6). (B) Pie charts displayed the distribution of differential metabolites by Super Class. (C) Heat map of differential lipids and lipid‐like molecules in METTL3 knockdown group. Red dots, upregulated metabolites; blue dots, downregulated metabolites; red arrow, three cyPGs. (D) Histogram showed eicosanoids which were differentially expressed between control group and METTL3 knockdown group. (E) KEGG pathway analysis was conducted to predict the altered signaling pathways following METTL3 knockdown. (F) GSEA pathway analysis from RNA‐Seq data showed an inverse correlation between oxidative phosphorylation and METTL3 knockdown. (G) Box plots showed the levels of glutathione and S‐(PGJ2)‐glutathione were differentially expressed between control group and METTL3 knockdown group. (H) ATP production and maximal respiration of HLECs were determined based on oxygen consumption rate (OCR). *p < 0.05 versus Ctrl, ^#^ *p* < 0.05 versus PGA2 or PGA2+GSH.

Under pathological conditions, abnormal angiogenic or lymphangiogenic effects require a sufficient energy supply.^20^ To understand the inhibitory mechanism of METTL3–lecKO on lymphangiogenesis, we conducted untargeted metabolomics profiling to compare metabolomics profiling between HLECs transfected with METTL3 shRNA or negative control shRNA. The results revealed that 412 differential metabolites were identified, including 289 upregulated metabolites and 123 downregulated metabolites (Figure 4A). To display the classification of these differential metabolites, the pie charts were drawn for Class (Figure S6A), Super Class (Figure 4B), and Sub Class (Figure S6B), respectively.

Hierarchical cluster analysis revealed that most of differential metabolites were fatty acyls‐lipids and lipid‐like molecules (Figure 4C). PGs are known as important regulators of inflammatory response, pain, and fever.^21^, ^22^ In PG family, there are a special class of eicosanoid acids called cyclopentenone prostaglandins (CyPGs), which contain a cyclopentenone ring structure with a highly reactive α, β‐unsaturated carbonyl group (Figure S6C). CyPGs mainly include PGs A and J series, their metabolites and PG‐like compounds, and isoprostaglandins (IsoPs). CyPGs can inhibit inflammatory response, cell growth, angiogenesis, and viral infection by targeting transcription factors and signal transduction pathway.^23^, ^24^ Herein, METTL3 knockdown caused enhanced levels of IsoPs and cyPGs (Figure 4D), suggesting that METTL3 regulates PG metabolism in HLECs.

We then carried out KEGG analysis to determine which metabolomic pathways were altered following METTL3 knockdown. We selected the altered pathways (*p < *0.05) for scatter plot displaying and observed oxidative phosphorylation (OXPHOS) signaling appeared at the downregulated processes (Figure 4E). We also conducted RNA‐Seq transcriptome analysis to determine the effects of METTL3 knockdown on gene expression in HLECs. Gene set enrichment analysis (GSEA) revealed an inverse correlation between OXPHOS and METTL3 knockdown (Figure 4F). GSH is a major endogenous thiol antioxidant and plays a key role in maintaining cellular redox balance and detoxification of exogenous and endogenous compounds.^24^ METTL3 knockdown led to decreased levels of GSH and increased levels of S‐(PGJ2)–glutathione (GSH) conjugates (formed by Michael addition of cyPGs to GSH) (Figure 4G), indicating enhanced oxidative stress. Moreover, GSEA pathway analysis revealed an inverse correlation between METTL3 expression and GSH metabolism (Figure S7).

Oxygen consumption rate (OCR) is a critical indicator of mitochondrial OXPHOS efficiency.^25^ To determine the effect of cyPGs administration on mitochondrial respiration, we measured OCR using the Seahorse XF extracellular flux analyzer in HLECs. Due to its ease of access, PGA2 was chosen as the representative cyPG. Administration of PGA2 inhibited ATP production and maximal respiration, while this inhibition was partially interrupted by GSH addition (Figure 4H). Collectively, these results show that METTL3 regulates PG metabolism and OXPHOS in HLECs.

### 2.5 METTL3 regulates PG metabolism by targeting PTGS2/COX‐2 pathway

**FIGURE 5:**
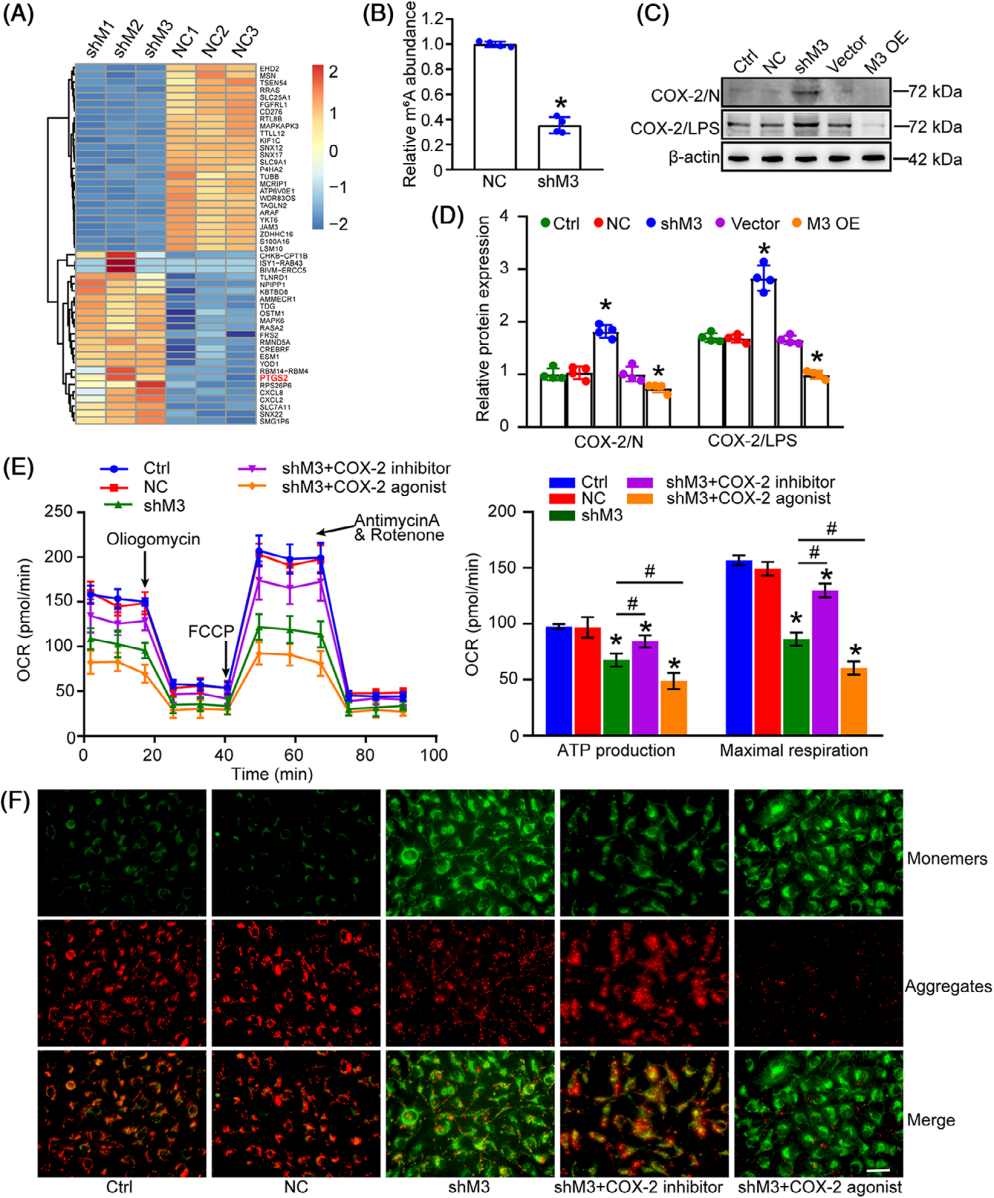
METTL3 regulates prostaglandin metabolism and oxidative phosphorylation by targeting PTGS2/COX‐2 pathway. (A) Heatmap and clustering of partial differentially expressed genes detected by RNA sequencing in HLECs between negative control group (NC) and METTL3 knockdown (shM) group. Blue indicates decreased level; red indicates increased level. (B) The levels of m^6^A RNA modification in the specific regions of PTGS2 gene were detected by colorimetric quantification (*n* = 4; *p < 0.05 vs. NC; Student's *t*‐test). (C and D) Western blot analysis was performed to assess COX‐2 protein levels in HLECs following METTL3 manipulation. β‐Actin served as a loading control. Densitometric quantification of immunoblots revealed significant differences between groups (*n* = 4, *p < 0.05 vs. Ctrl, one‐way ANOVA with Bonferroni test). (E) ATP production and maximal respiration of HLECs were detected based on oxygen consumption rate (OCR). *p < 0.05 versus Ctrl; ^#^ *p* < 0.05 between the marked groups. (F) Representative fluorescence images of MMPs following JC‐1 incubation. Red, JC‐1 aggregates in healthy mitochondria; green, JC‐1 monomer indicating MMP dissipation; Scale bar, 20 µm.

To elucidate the mechanisms underlying METTL3 regulation of PG metabolism, we performed cluster analysis on differentially expressed genes identified by RNA sequencing. Among these dysregulated genes, we found that PTGS2 (PG–endoperoxide synthase 2), a gene related to PG metabolism, was differentially expressed between METTL3 knockdown group and control group (Figure 5A). PTGS2, also known as cyclooxygenase 2 (COX‐2), is an enzyme involved in PG biosynthesis, which is responsible for prostate‐like biosynthesis during inflammation and mitogenesis. METTL3 knockdown resulted in impaired m^6^A abundance of PTGS2, suggesting that PTGS2 is a potential target of METTL3 (Figure 5B). Then, we investigated the effects of METTL3 knockdown on PTGS2 expression in HLECs. Western blot assays revealed that METTL3 knockdown led to increased levels of PTGS2‐encoded COX‐2 protein. By contrast, METTL3 overexpression led to reduced levels of COX‐2 protein. Similar results were also observed following LPS treatment (Figure 5C,D). Next, we measured OCR of HLECs under normal condition and inflammatory condition. METTL3 knockdown led to reduced levels of OCR. Selective COX‐2 inhibitor (lumiracoxib) interrupted the reduction of OCR, while COX‐2 agonist (COX‐2 mix its substrate arachidonic acid, AA) further aggravated METTL3 knockdown‐induced reduction of OCR (Figure 5E). Similar results were also observed following LPS treatment (Figure S8). JC‐1 dye was used to detect mitochondrial membrane potential (MMP). METTL3 knockdown led to reduced MMP. COX‐2 inhibitor could reverse the reduction of MMP, while COX‐2 agonist further aggravated METTL3 knockdown‐induced reduction of MMP (Figure 5F). Collectively, the results suggest that METTL3 regulates PG metabolism by targeting PTGS2/COX‐2 pathway.

### 2.6 Administration of PG metabolite, PGA2, interrupts METTL3 overexpression‐induced lymphangiogenic endothelial activation

**FIGURE 6:**
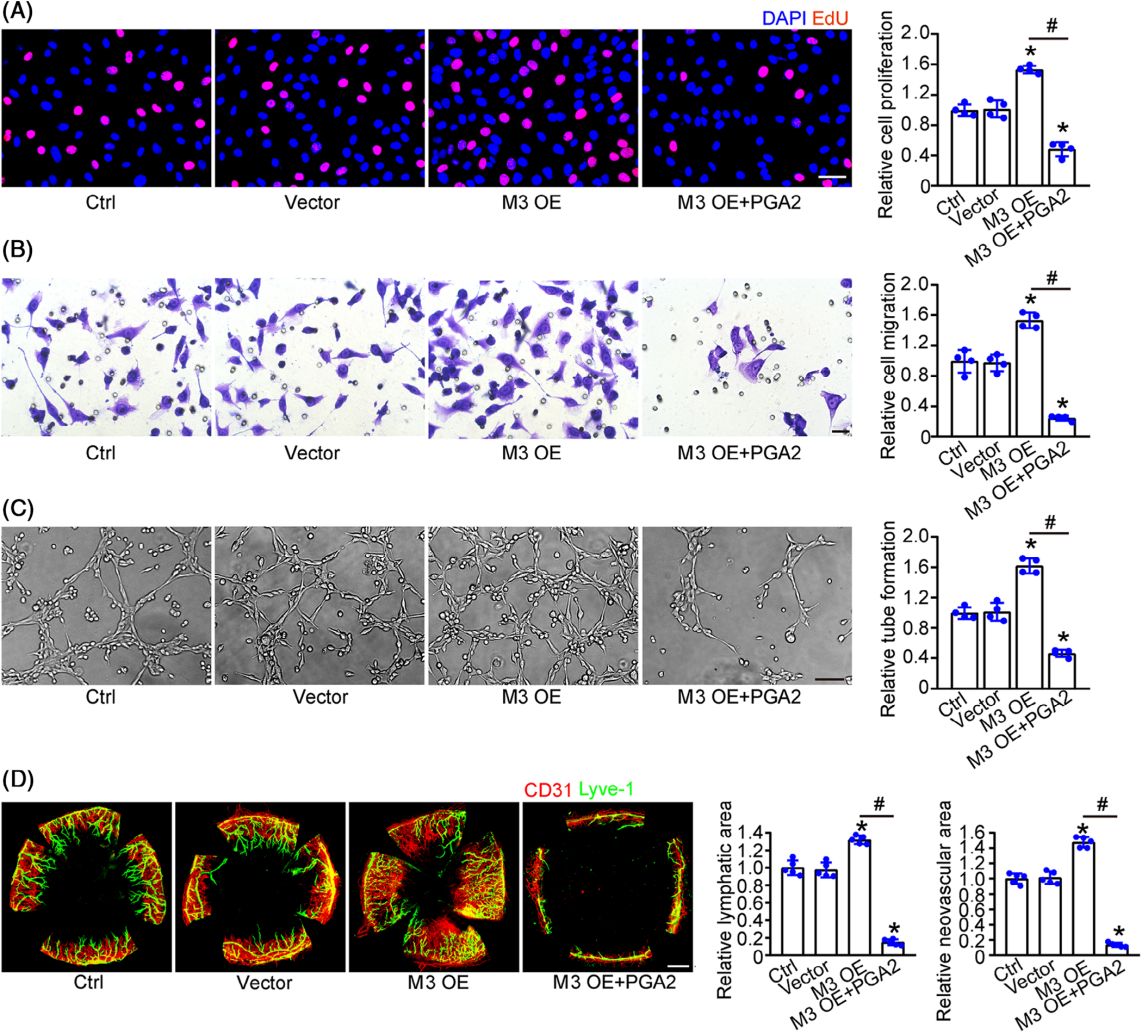
Administration of prostaglandin metabolite, PGA2, interrupts METTL3 overexpression‐induced lymphangiogenic endothelial activation. (A–C) HLECs were transfected with null vector (vector), pcDNA3.1–METTL3 vector (M3 OE), cDNA3.1–METTL3 vector (M3 OE) with PGA2 addition, or left untreated for 24 h (Ctrl). EdU detection kit and quantification analysis was conducted to detect cell proliferation. Blue, DAPI; red, EdU; scale bar, 20 µm (A). Transwell assay and quantification analysis was conducted to detect cell migration. Scale bar, 20 µm (B). HLECs were seeded on the Matrigel matrix and the tube‐like structures were observed 6 h after cell seeding. Average tube length for each field was statistically analyzed. Scale bar, 50 µm (C). *n* = 4; *p < 0.05 versus. Ctrl, #*p* < 0.05 M3 OE versus M3 OE+PGA2; one‐way ANOVA with Bonferroni test. (D) C57BL/6 mice were stitched with 10‐0 nylon sutures and injected with null vector (vector), METTL3 overexpression virus (M3 OE), METTL3 overexpression virus (M3 OE) with PGA2 addition or left untreated (Ctrl). After day 7 following treatment, the corneas were harvested and flat‐mounted. Then, they were stained with CD31 (red) to show corneal neovascularization and LYVE‐1 (green) to show corneal lymphangiogenesis. Scale bar, 200 µm; *n* = 5; Mann–Whitney *U*‐test with Bonferroni test.

To investigate the role of PG metabolite in lymphangiogenesis, we first performed the replenishment experiments in HLECs. METTL3 overexpression led to increased proliferation ability, migration ability, and tube formation ability of HLECs. Administration of a PG metabolite, PGA2, could reverse this trend and inhibit the proliferation, migration, and tube formation ability of HLECs induced by METTL3 overexpression (Figure 6A–C). In the sutured corneas, METTL3 overexpression contributed to corneal lymphangiogenesis and neovascularization, while administration of PGA2 could interrupt METTL3 overexpression‐induced corneal lymphangiogenesis and neovascularization (Figure 6D). These results suggest that METTL3 regulates pathological lymphangiogenesis via PG metabolism.

### 2.7 METTL3 knockdown enhances PTGS2 mRNA stability via YTHDF2‐dependent pathway

**FIGURE 7:**
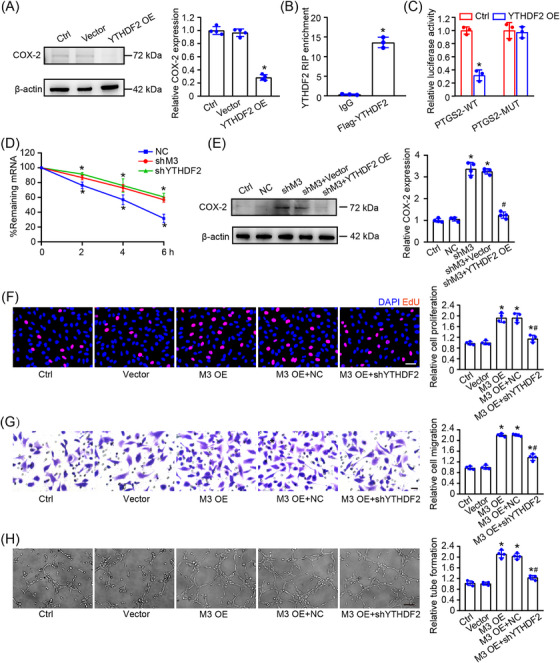
METTL3 knockdown enhances PTGS2 mRNA stability via YTHDF2‐dependent pathway. (A) Western blot analysis was performed to assess COX‐2 protein levels in HLECs transfected with null vector, Flag‐YTHDF2 plasmid, or left untreated for 24 h. β‐Actin served as a loading control. Densitometric quantification of immunoblots revealed significant differences between groups (*n* = 4, *p < 0.05 vs. Ctrl, one‐way ANOVA with Bonferroni test). (B) RIP analysis of the interaction of PTGS2 with YTHDF2 in HLECs transfected with Flag‐YTHDF2 plasmid. Enrichment of PTGS2 was determined by qPCR assays and normalized to the input (*n* = 3, Student *t*‐test). (C) Relative luciferase activity of wild‐type (WT) PTGS2‐3′ UTR or mutant (MUT) PTGS2‐3′ UTR luciferase reporter in HLECs transfected with null vector (Ctrl) or Flag‐YTHDF2 plasmid. Luciferase activity was detected at 36 h following transfection (*n* = 3, Student *t*‐test). (D) HLECs were treated as shown. Two days after transfection, HLECs were treated with Actinomycin D (Act D; 5 μg/mL) for the indicated time points. PTGS2 mRNA stability was assessed by qRT‐PCR, normalized to β‐actin mRNA levels. Initial mRNA levels were set to 100% (*n* = 4, Mann–Whitney *U*‐test with Bonferroni test). (E) HLECs were treated as shown. Western blots were conducted to detect the levels of COX‐2 protein (*n* = 4). *p < 0.05 versus Ctrl, #*p* < 0.05 shM3 versus. shM3+ YTHDF2 OE; one‐way ANOVA with Bonferroni test. (F–H) HLECs were transfected with null vector, Flag‐METTL3 plasmid (M3 OE), with or without YTHDF2 shRNA, or left untreated (Ctrl) for 24 h. EdU detection kit and quantification analysis was conducted to detect cell proliferation. Blue, DAPI; red, EdU; scale bar, 20 µm (F). Transwell assays and quantification analysis was conducted to detect cell migration. Scale bar, 20 µm (G). Tube formation assays and quantification analysis was conducted to detect tube formation ability (H). Scale bar, 50 µm; *n* = 4; *p < 0.05 versus Ctrl, #*p* < 0.05 M3 OE versus M3 OE+ shYTHDF2; one‐way ANOVA with Bonferroni test.

We next studied the mechanism how METTL3‐mediated m^6^A modification altered the expression of PTGS2 in HLECs. m^6^A is often recognized by specific m^6^A‐binding proteins to play their roles. m^6^A methylation is inversely correlated with PTGS2 expression. Given the m^6^A‐binding protein, YTH domain family 2 (YTHDF2), can bind to mRNAs and degrade mRNAs, we thus hypothesized that PTGS2 mRNA may be recognized and degraded by YTHDF2.^26^ qRT‐PCR assays showed that PTGS2 mRNA level decreased in response to YTHDF2 overexpression, but not YTHDF1 or YTHDF3 (Figure S9). As expected, overexpression of YTHDF2 led to reduced levels of COX‐2 protein in HLECs (Figure 7A). RNA immunoprecipitation followed by qPCR analysis further verified that PTGS2 was a target gene of YTHDF2 (Figure 7B). Dual luciferase assays revealed that ectopic YTHDF2 significantly decreased the luciferase activity in the reporter carrying wild‐type PTGS2 3′‐UTR fragment but not in the reporter carrying mutant PTGS2 3′‐UTR fragment in m^6^A consensus sites (Figure 7C). Detection of PTGS2 mRNA decay following RNA synthesis inhibition with Actinomycin D revealed that knockdown of YTHDF2 or METTL3 led to increased stability of PTGS2 mRNA (Figure 7D), suggesting that YTHDF2 destabilized PTGS2 mRNA in an m^6^A‐dependent manner. Moreover, YTHDF2 overexpression could interrupt the enhancement of COX‐2 protein levels induced by METTL3 knockdown in HLECs (Figure 7E). YTHDF2 knockdown could interrupt the enhancement of proliferation, migration, and tube formation ability of HLECs induced by METTL3 overexpression (Figure 7F–H). Taken together, these results suggest that METTL3 regulates PTGS2 mRNA stability in an YTHDF2‐dependent manner.

### 2.8 Pharmacological inhibition of METTL3 suppresses pathological lymphangiogenesis

**FIGURE 8:**
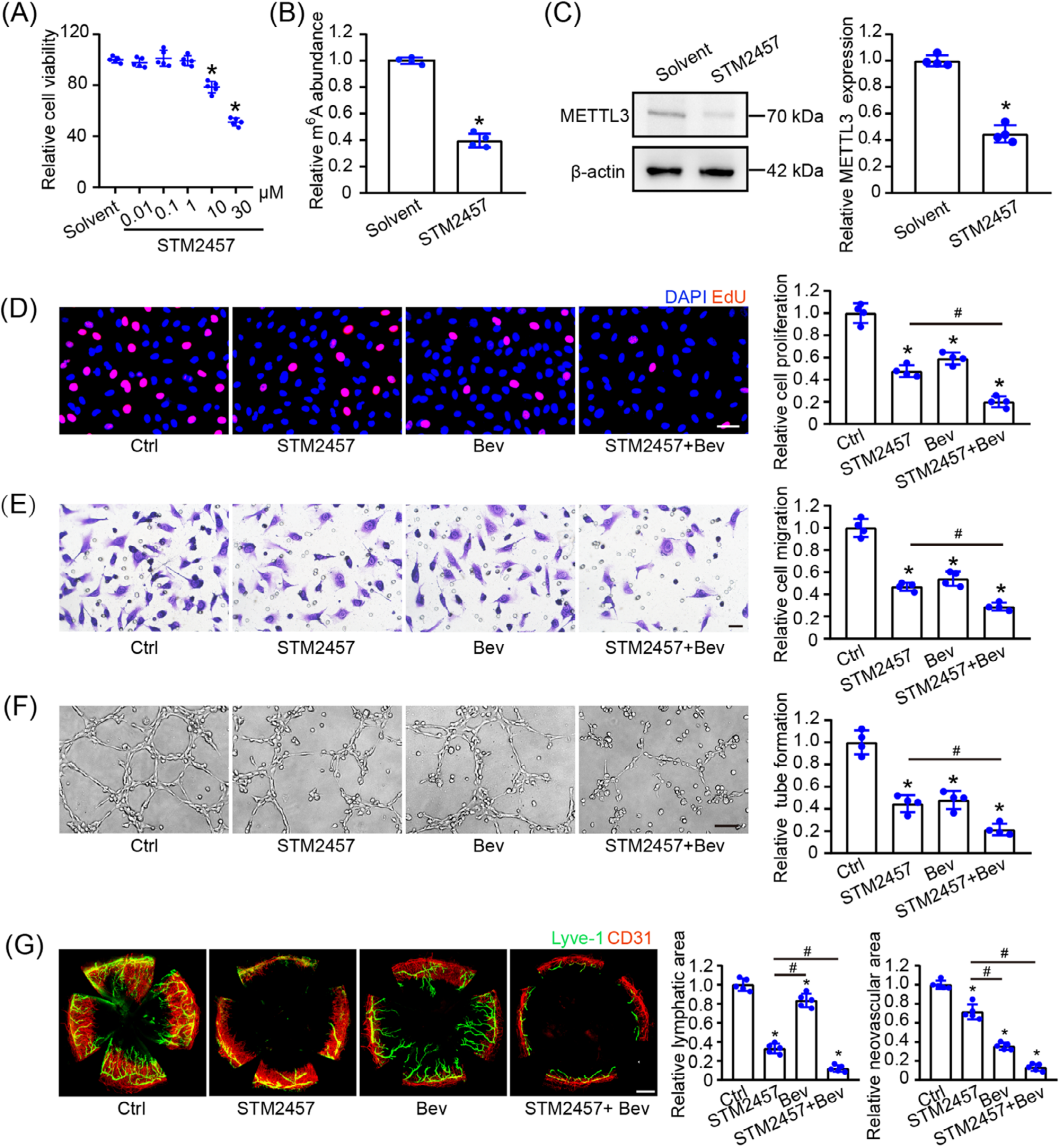
Pharmacological inhibition of METTL3 suppresses pathological lymphangiogenesis. (A) HLECs were treated with different concentrations of STM2457 for 24 h. CCK‐8 assays were performed to detect cell viability (*n* = 5, *p < 0.05 vs. Solvent, one‐way ANOVA with Bonferroni test). (B and C) HLECs were treated with solvent or 1 μM STM2457. m^6^A RNA modification levels were assessed by colorimetric quantification (B). Western blot analysis with densitometric quantification determined METTL3 protein levels, with β‐actin as a loading control (C). *n* = 4, *p < 0.05 versus Solvent, one‐way ANOVA with Bonferroni test. (D–F) HLECs were treated with STM2457 (1 μM), bevacizumab (Bev, 10 μg/mL), Bev plus STM2457 (1 μM), or left untreated (Ctrl) for 24 h. EdU assays and quantification analyses were performed to detect cell proliferation. Blue, DAPI; red, EdU; scale bar, 20 µm (D). Transwell assays and quantification analyses were performed to detect cell migration. Scale bar, 20 µm (E). Tube formation assays and quantification analyses were performed to detect tube formation capacity. Scale bar, 50 µm (F). *n* = 4; *p < 0.05 versus Ctrl, ^#^ *p *< 0.05 between the marked groups; one‐way ANOVA with Bonferroni test. (G) C57BL/6 mice were stitched with 10‐0 nylon sutures and injected with STM2457 (1 μM), bevacizumab (Bev; 10 mg/mL), Bev plus STM2457 (1 μM), or left untreated (Ctrl) for 7 days. The corneas were harvested and flat‐mounted. They were stained with CD31 (blood vessels) and LYVE‐1 (lymphatic vessels) for immunofluorescence analysis (scale bar, 200 µm). Lymphangiogenesis and neovascularization areas were quantified and compared across groups (*n* = 5; *p < 0.05 vs. Ctrl, ^#^ *p *< 0.05 between the marked groups; Mann–Whitney *U*‐test with Bonferroni test).

To determine the potential of METTL3 as a therapeutic agent for pathological lymphangiogenesis, a potent and selective inhibitor of METTL3, STM2457, was used to abolish the catalytic function of METTL3. We first determined the effects of STM2457 administration on the viability of HLECs in vitro. CCK‐8 (Cell Counting Kit ‐ 8) assays revealed that STM2457 administration had no obvious cytotoxicity up to 1 μM (Figure 8A). METTL3 inhibition by STM2457 administration led to reduced m^6^A levels and decreased expression of METTL3, suggesting that STM2457 treatment inhibits m^6^A mRNA methylation (Figure 8B,C). Both STM2457 and bevacizumab suppressed HLEC proliferation, migration, and tube formation. The combination of STM2457 and bevacizumab exhibited enhanced antiangiogenic effects (Figure 8D‐F).

To determine the effects of STM2457 administration on pathological lymphangiogenesis in vivo, STM2457 and bevacizumab were injected into the subconjunctiva of the sutured corneas, respectively. However, STM2457 exhibited superior antilymphangiogenic efficacy compared with bevacizumab, while bevacizumab demonstrated greater antiangiogenic potency. The combination of STM2457 and bevacizumab yielded optimal therapeutic outcomes for both lymphangiogenesis and angiogenesis (Figure 8G). Collectively, these findings highlight STM2457 as a promising therapeutic candidate for targeting pathological lymphangiogenesis.

## 3 DISCUSSION

m^6^A is a prevalent mRNA modification, which has obtained great interests due to its broad implications in various biological processes and diseases.^27^, ^28^ METTL3 is a key methyltransferase for m^6^A methylation.^29^ This study unveils a novel function for METTL3‐mediated m^6^A modification in promoting pathological lymphangiogenesis. Knockdown of METTL3 suppresses corneal sutures‐induced lymphangiogenesis and subcutaneous graft‐induced intratumoral lymphangiogenesis, suggesting its potential as a therapeutic target for lymphangiogenesis‐related diseases.

m^6^A RNA modification is a reversible process regulated by methyltransferase, demethylase, and m^6^A‐binding proteins (readers).^21^, ^30^ We show that inflammatory stress upregulated METTL3. By contrast, the levels of other m^6^A regulatory factors remained unchanged. While METTL3 has been implicated in various diseases, its role in lymphangiogenesis was previously unknown.^29^ This study unveils METTL3 as a critical regulator of pathological lymphangiogenesis. Inhibition of METTL3 suppresses lymphatic vessel growth in both corneal and tumor models, expanding our understanding of its function and highlighting its therapeutic potential.

Normal cornea has no lymphatic vessels and blood vessels. Corneal avascularity is disrupted by angiogenesis and lymphangiogenesis.^32^ Solid tumors often necessitate the development of new blood and lymphatic vessels, highlighting the role of angiogenesis and lymphangiogenesis in tumorigenesis.^33^ Normal lymphatic vessels contribute to inflammatory responses, immune regulation, and surveillance by controlling plasma volume, tissue pressure, and leukocyte trafficking. Growth factors and cytokines secreted by stromal and inflammatory cells influence both angiogenesis and lymphangiogenesis.^34^, ^35^ Our findings demonstrate that inflammatory stress and corneal suture injury induce elevated METTL3‐mediated m^6^A modification. Conditional lymphatic Mettl3 knockout effectively inhibits corneal and tumor‐associated lymphangiogenesis, suggesting a potential role for antilymphangiogenic therapies alongside antiangiogenic approaches. The intricate relationship between vascular and lymphatic systems emphasizes the potential benefits of combination therapies targeting both vessel types for diseases characterized by abnormal vessel growth.

Corneal and tumor tissues exhibit cellular heterogeneity in terms of genetic and epigenetic profiles.^36^, ^37^ To sustain rapid proliferation, cells undergoing lymphangiogenesis or angiogenesis must adapt to hypoxic and nutrient‐deficient microenvironments and undergo metabolic reprogramming.^38^ The inflammatory microenvironment, characterized by oxidative stress and lipid peroxidation, is closely linked to vascular diseases. Inflammatory cytokines and reactive oxygen species mutually amplify, contributing to disease progression.^39^, ^40^ Lipid peroxidation is a key feature of oxidant stress.^41^ We observed that METTL3 knockdown alters the cellular lipidome, suggesting its involvement in lipid metabolism. Inflammation induces lipid peroxidation through ROS generation, leading to the formation of oxidized lipid products. These products contribute to cellular stress by overwhelming antioxidant capacity.

A significant group of bioactive lipids, including PGs, IsoPs, and leukotrienes, are generated from arachidonic acid oxidation during inflammatory responses. PGs primarily mediate inflammatory responses, pain, and fever.^21^, ^22^ Among different types of PGs, cyPGs with a cyclopentenone ring structure have the potent anti‐inflammatory, antitumor, and antiviral activities. CyPGs exert their effects by inhibiting cell cycle, apoptosis activation, stress response, and inhibiting protein synthesis.^42^ Our findings suggest that METTL3 serves as a therapeutic target for inflammatory diseases characterized by dysregulated PG metabolism. Modulating METTL3 levels offer a strategy to alter the amount of oxidized lipid products and attenuate inflammatory responses.

Lymphangiogenesis requires a substantial energy supply to transition HLECs from quiescence to a proliferative, migratory phenotype. Mitochondrial OXPHOS is essential for ATP production, while mitochondrial dysfunction can impair lymphatic endothelial function.^9^, ^43^, ^44^ GSH, a key antioxidant, maintains cellular redox balance and influences OXPHOS. CyPGs, reactive electrophilic compounds, readily bind to GSH. METTL3 knockdown elevates cyPG levels, depletes GSH, and consequently impairs mitochondrial OXPHOS, leading to lymphatic endothelial dysfunction. We also identified PTGS2 (COX‐2), an enzyme catalyzing PG synthesis from arachidonic acid, as a potential downstream target of METTL3. PTGS2 is implicated in lipid oxidation pathways associated with inflammation and cell proliferation.^45^, ^46^ PTGS2 expression is regulated by METTL3‐mediated m^6^A modification through an YTHDF2‐dependent mechanism. Increased METTL3 levels correlate with decreased PTGS2 expression, suggesting a potential role in modulating lymphatic endothelial cell behavior.

Inhibiting of lymphangiogenesis has profound clinical implications. In ophthalmology, corneal transplantation highlights the detrimental effects of lymphatic vessel growth on graft survival, as immune rejection is often associated with lymphangiogenesis.^47^ Moreover, lymphangiogenesis contributes to dry eye syndrome and corneal inflammation.^48^ Beyond ocular diseases, lymphangiogenesis is critical in tumor metastasis and progression.^49^ The interconnectedness of the vascular and lymphatic systems highlights the potential of targeting lymphangiogenesis for treating cardiovascular, neurological, and other diseases.^50^, ^51^

Our study still has several limitations. Lymphatic system remains less characterized compared with vascular system, and challenges persist in visualizing and studying early‐stage lymphangiogenesis. While cell line and animal models provide valuable insights, they may not fully recapitulate the complexity of human lymphangiogenesis, particularly in disease contexts. Moreover, in vivo imaging limitations hinder precise tracking and quantification of lymphangiogenesis. The intricate interplay between lymphatic, immune, and vascular systems introduces additional complexities in dissecting the underlying mechanism.

In conclusion, this study elucidates a critical role for METTL3‐mediated m^6^A modification in pathological lymphangiogenesis. METTL3 inhibition suppresses lymphatic vessel growth in both corneal and tumor models. Mechanistically, METTL3 regulates lymphangiogenesis and angiogenesis via PG metabolism. These findings establish METTL3 as a promising therapeutic target for lymphangiogenesis‐related diseases.

## 4 MATERIALS AND METHODS

### 4.1 Clinical sample collection

During corneal transplantation surgery, the affected corneas were removed. These excised fragments were then collected for further analysis, such as bacterial/fungal cultures or pathological diagnosis. Any remaining corneal tissue suitable for clinical use was also collected. Patients included in the study met the following criteria: required corneal transplantation due to bacterial or viral keratitis or required corneal transplantation due to corneal scarring caused by trauma or chemical injury.

### 4.2 Animals

C57BL/6 mice were purchased from Nanjing Junke Bioengineering Corporation, Ltd. (Nanjing, China). Mettl3^flox/flox^ mice and Lyve1–IRES–iCre mice were purchased from GemPharmatech (Nanjing, China). Mettl3^flox/flox^ mice were crossed with Lyve1–IRES–iCre mice to generate Mettl3–lecKO mice. All mice were housed at a constant temperature of 25°C and relative humidity of 60% under pathogen‐free conditions, with unrestricted access to water and food.

### 4.3 Corneal suture model

Anesthesia for the mice involved a combination of xylazine (10 mg/kg) and ketamine (100 mg/kg; Aibei Biotechnology; M2920) administered systemically. Additionally, topical anesthesia was achieved with isobucaine hydrochloride (Santen, Japan). 10‐0 Nylon sutures (Huawei, China) were individually placed 2 mm from the limbus within corneal stroma, avoiding penetration into anterior chamber. To stimulate corneal neovascularization and lymphangiogenesis, three sutures were positioned 120° apart around corneal circumference. Finally, antibiotic ointment was applied to minimize postoperative infection.

### 4.4 Cell culture

HLECs were cultured in Endothelial Cell Basal Medium MV (PromoCell; C‐22220) supplemented with 10% fetal bovine serum (ScienCell; 0500), 100 U/mL penicillin, and 100 μg/mL streptomycin (Gibco; 15140122) at 37°C with 21% O_2_ and 5% CO_2_. All experiments were performed before passage 6.

### 4.5 m^6^A dot blot assay

Total RNAs were extracted from HLECs with TRIzol reagent (Invitrogen; 15596026). The samples were spotted onto the Amersham Hybond‐N^+^ membranes (GE Healthcare; 45‐000‐763) and UV cross‐linked. Then, the membranes were incubated with m^6^A antibody (1:1000; Synaptic Systems; 202003) and detected by chemiluminescence with the Clarity™ Western ECL Substrate. RNA density of the dot was quantified by Image J.

### 4.6 Measurement of mitochondrial oxygen consumption

HLECs were seeded at a density of 4 × 10^4^ cells per well on Seahorse XFe96 V3 PS microplates coated with 0.1% (w/v) gelatin (Agilent Technologies; 103799‐100). OCR was measured using an Agilent Seahorse XFe96 Analyzer. The analyzer generated OCR curves following sequential injections of oligomycin (1 μmol/L), FCCP (1.5 μmol/L), and a mixture of antimycin A (1 μmol/L) and rotenone (1 μmol/L). A final manual inspection of all wells was performed using a light microscope

### 4.7 Statistical analysis

Data are presented as mean ± SEM. For normally distributed data with equal variances, we employed Student's *t*‐test for two‐group comparisons and one‐way analysis of variance (ANOVA) followed by Bonferroni's posthoc test for multigroup comparisons. For non‐normally distributed data or data with unequal variances, the Mann–Whitney *U*‐test was used for two‐group comparisons, while Kruskal–Wallis test followed by Bonferroni's posthoc test was used for multigroup comparisons. *p *< 0.05 was considered statistically significant.

## AUTHOR CONTRIBUTIONS

**Biao Yan and Qin Jiang**: designed the research. **Lianjun Shi; Shuting Lu; Xue Han; Fan Ye; Xiumiao Li; and Ziran Zhang**: performed the experiments. **Lianjun Shi; Shuting Lu; and Xue Han**: analyzed the data. **Lianjun Shi and Biao Yan**: wrote and revised the article. All authors have read and approved the final manuscript.

## CONFLICT OF INTEREST STATEMENT

The authors declare no conflict of interest.

## ETHICS STATEMENT

Animal experiments were approved by the Animal Experiment Management Committee of the author's institute (Ethics Committee No. 2019‐02‐20‐66). The animals were performed according to ARVO Statement for the use of animals for ophthalmic Research. The surgical specimens were treated according to the Declaration of Helsinki. The involved patients obtained the informed consent before inclusion (Ethics Committee No. NJMEH‐2020‐1021).

## Supporting information

Supporting Information

## Data Availability

Raw RNA sequencing data have been submitted to Gene Expression Omnibus datasets with accession number GSE273930. Raw metabolomics data are available from the MetaboLights repository under the accession number MTBLS 10765. The authors declare that other data supporting the findings of this study are available in the manuscript.
