# Supplementary material for: Lymphatic‐specific methyltransferase‐like 3‐mediated m6A modification drives vascular patterning through prostaglandin metabolism reprogramming
Source: MedComm (2020). 2024 Oct 4;5(10):e728. doi: 10.1002/mco2.728 (PMC11450254; doi:10.1002/mco2.728)
Supplement: Supplementary file 1 — Supporting Information [file MCO2-5-e728-s001.pdf]

Supplemental data

Supplemental methods

Immunofluorescence assay

The fresh corneas were harvested at day 7 day following sutures and were fixed in 4% paraformaldehyde for 0.5 h at room temperature. Then, the corneas were blocked with 10% bovine serum albumin (BSA) with 1% Triton X-100. To observe corneal lymphangiogenesis and neovascularization, the corneas were incubated with LYVE-1 (1:100, Abcam, ab14917) and CD31 (1:100, BD Biosciences, 550274) primary antibody overnight at 4°C and stained by goat anti-rabbit IgG (H+L) cross-adsorbed secondary antibody, Alexa Fluor™ 594 (1:200, Invitrogen, A11012) and goat anti-rat IgG (H+L) cross-adsorbed secondary antibody, Alexa Fluor™ 488 (1:200, Invitrogen, A11006) secondary antibody for 2 h at room temperature. The images were captured by a fluorescence microscope (Olympus, Japan) and Image J software was used to detect the lymphangiogenic and angiogenic areas.

In vivo xenografts model

U87 MG cells (5×10^6, Procell, CL-0238) were implanted into the subcutaneous axilla on one side of low abdomen of the mice. Bevacizumab was injected weekly around the subcutaneous masses. Tumor size was measured weekly and recorded. Then, the tumors were excised from the euthanized animals at day 35 after injection and used for immunofluorescence staining.

Quantification of m^{6}A RNA level

The levels of m^{6}A RNA modification were determined by an EpiQuik m^{6}A RNA Methylation Quantification Kit (EpiGentek, P-9008-48). In brief, total RNA (100 ng) was conjugated to each well followed by the addition of capture antibody solution and detection antibody solution. m^{6}A modification levels were quantified by absorbance measurements at 450 nm. All samples were analyzed in triplicate using standard curves to determine m6A RNA concentrations.

Quantitative real-time PCR

Total RNA was extracted using TRIzol reagent (Invitrogen, 15596026). cDNA synthesis was performed using specific primers and SYBR Green Master Mix (Thermo Fisher Scientific, A25741). qRT-PCR was conducted on a 7500 Fast Real-Time PCR system (Thermo Fisher Scientific, USA) to determine relative gene expression using the $2^{-\Delta\Delta Ct}$ method.

Western blot

The cells or tissues were lysed using the RIPA buffer (Beyotime, P0013B) supplemented with the protease inhibitor cocktail (Roche, 4693132001) to obtain total proteins. The proteins were separated via SDS-PAGE gels and then transferred to PVDF membranes (Sigma-Aldrich, IPVH00010). After blocking the membranes with 5% BSA (BioFroxx, 4240GR100) for 1 h, the membranes were indicated with the specific primary antibody at 4°C overnight. After washing with PBST (Biosharp, BL314B) three times, the membranes were incubated with the secondary antibody for 3 h at room temperature. BCA Protein Quantitation Kit (Beyotime, P0012) was used

to quantify the amount of proteins. The bands were visualized by the enhanced chemiluminescence detection (Tanon, China). The primary antibodies were shown below: ALKBH5 (Proteintech, 16837-1-AP), WTAP (Proteintech, 60188-1-Ig), FTO (proteintech, 27226-1-AP), METTL3 (Proteintech, 15073-1-AP), METTL14 (Proteintech, 26158-1-AP), IL-1β (Proteintech, 66737-1-Ig), COX2/PTGS2 (ABclonal, A3560), GAPDH (Proteintech, 60004-1-Ig), and β-Actin (Proteintech, 66009-1-Ig). The secondary antibodies were shown below: horseradish peroxidase labeled goat anti-mouse IgG (H+L) (Beyotime, A0216) and horseradish peroxidase labeled goat anti-rabbit IgG(H+L) (Beyotime, A0208).

RIP-qPCR assay

Total RNAs were extracted using the TRIzol reagent (Invitrogen, Carlsbad, CA, United States) and were sonicated into 100-150 nt fragments. The fragments were incubated with m^6A antibody (Synaptic Systems, 202003) for immunoprecipitation (IP). m^6A RNA enrichment was determined by qRT-PCR assays. Briefly, 4 μg of fragmented mRNAs was incubated with 2 μg of m^6A antibody or rabbit normal immunoglobulin G (IgG, Negative control) in 1 × IP buffer (10 mM Tris-HCl, pH 7.4, 150 mM NaCl, 0.1% Nonidet P-40 [NP-40]) for 2 h at 4 °C. Then, m^6A-IP mixture was combined with Dynabeads protein A (Life Technologies; 10002D), washed three times with 1 × IP buffer, and washed twice with 1 × wash buffer (10 mM Tris-HCl, pH 7.4, 50 mM NaCl, 0.1% NP-40). The bound RNAs were eluted with the elution buffer (10 mM Tris-HCl, pH 7.4, 1 mM EDTA, 0.05% SDS, 40 U proteinase K) at 50 °C for 30 min and purified. m^6A methylation levels of target genes were detected by

qPCRs after m^6A-IP.

MTT assay

Cell viability was measured by MTT [3-(4, 5-dimethylthiazol-2-yl)-2, 5-diphenyltetrazolium bromide, Thermo Fisher Scientific, M6494] assay. HLECs were incubated with MTT solution for 4 h, followed by DMSO treatment to solubilize the formazan product. The absorbance was determined at 490 nm wavelength and analyzed by a microplate reader (Molecular Devices, USA).

Cell migration assay

Cell migration was determined by transwell assay. Polycarbonate transwell filters with 8.0-μm pores were inserted into the lower chambers. A total of 5×10^4 cells, suspended in 2% FBS, were plated in the insert chamber, which was supplemented with 600 μL of 10% FBS medium. After 12 h of culture, the cells were fixed and permeabilized in propyl alcohol for 15 min and then stained with 5% crystal violet for 10 min at 4°C. The number and morphology of the migrated cells were observed under an inverted microscope.

Tube formation assay

Matrigel solution was plated onto the pre-cooled 24-well plates and incubated for 30 min at 37°C. HLECs were then plated onto the Matrigel and incubated at 37°C for 6 h. The images were captured using an Olympus microscope (DB80, Tokyo, Japan).

EdU assay

Cell proliferation was detected by a Cell-Light EdU DNA Cell proliferation Kit (RiboBio, China). Briefly, HLECs were seeded onto a 24-well plate. Following the

required treatments, they were transfected and incubated with EdU at 37 °C for 4 h. 200 μL of Apollo 488 reaction cocktail (RiboBio, China) staining reaction solution was added to each well and incubated for 30 min. The nuclei were stained with DAPI (RiboBio, China, 1:1000) for 10 min. The images were examined by a fluorescence microscope (Olympus, Japan).

RNA-seq analysis

Total RNAs were isolated and purified using the TRIzol reagent (Invitrogen, 15596018) according to the manufacturer's protocol. Quality control was performed using NanoDrop ND-1000 (Thermo Fisher, USA). RNA integrity was detected and verified by Bioanalyzer 2100 (Agilent, USA) and electrophoresis with the denaturing agarose gel. Poly (A) RNA was purified from 1 μg of total RNAs using the Dynabeads Oligo (dT) 25-61005 (Thermo Fisher, USA). Then, the poly (A) RNA was fragmented into small pieces using the Magnesium RNA Fragmentation Module (NEB, E6150S). The cleaved RNA fragments were reversely transcribed into cDNAs by the SuperScript™ II Reverse Transcriptase (Invitrogen, 18064071), which were used to synthesize U-labeled second-stranded DNAs with *E. coli* DNA polymerase I (NEB, M0209S), RNase H (NEB, M0297S) and dUTP Solution (Thermo Fisher, R0133). A-base was added to each end enable ligation with an adaptor with a T-base at the end. Then, the fragment size was screened and purified with AMPure XP beads. After heat-labile UDG enzyme (NEB, M0280S) treatment of U-labeled second-stranded DNAs, the ligated products were amplified with PCRs to form a fragment size of 300 bp ± 50 bp library. Finally, we performed 2 × 150 bp paired-end

sequencing (PE150) on an illumina Novaseq™ 6000 (LC-Bio Technology CO., Ltd., China).

Clean reads were initially filtered using Cutadapt (version: cutadapt-1.9, https://cutadapt.readthedocs.io/en/stable/). Subsequently, all samples' reads were aligned to human reference genome (version: GRCh38, ftp://ftp.ensembl.org/pub/release-101/fasta/homo_sapiens/dna/) using HISAT2 (version: hisat2-2.2.1, https://daehwankimlab.github.io/hisat2/). The aligned reads for each sample were assembled using StringTie (https://ccb.jhu.edu/software/stringtie) with default settings. A comprehensive transcriptome was reconstructed by merging all transcriptomes using gffcompare (https://github.com/gpertea/gffcompare/). The expression levels were estimated using StringTie. Differentially expressed mRNAs were identified based on fold change > 2 or corrected $P < 0.05$ using parametric F-test comparing nested linear models implemented in the R package edgeR (https://bioconductor.org/packages/release/bioc/html/edgeR.html).

Dual luciferase activity assay

Expression vectors containing wild-type and mutant PTGS2 3' UTR with predicted YTHDF binding sites were constructed. HLECs were seeded into 24-well plates and co-transfected with YTHDF overexpression and either wild-type or mutant PTGS2 3' UTR luciferase reporter plasmids using Lipofectamine 6000. Luciferase activity was measured 24 h post-transfection using the Dual-Luciferase Reporter Assay System (Promega, E1910).

Measurement of mitochondrial membrane potential (MMP)

MMP was measured through tetraethylbenzimidazoly-lcarbocyanine iodide (JC-1) staining. Briefly, HLECs were seeded in a 24-well plate. After the required treatment, they were stained with JC-1 Assay kit (Beyotime, C2006) for 30 min according to the manufacturer's protocol. Then, HLECs were washed twice with PBS and observed by a fluorescence microscope (Olympus, Japan).

RNA stability assay

To assess mRNA stability, HLECs were treated with actinomycin D (5 μg/mL) to inhibit transcription. Relative mRNA levels were quantified at 0, 2, 4, and 6 h post-treatment. mRNA half-life was calculated based on mRNA decay rates, normalized to β-actin mRNA levels, with initial mRNA levels set at 100%.

LC/MS based untargeted metabolomics analysis

Cell samples were transferred to glass vials with the precooled methanol-water (V: V = 4:1). Then, chloroform was added and sonicated in an ice bath. The whole liquid was transferred to the centrifuge tube and 20 μL of tinternal standard (L-2-chlorophenylalanine) was added. The cells were sonicated in an ice- water bath for 20 min and left at -40 °C for 30 min. After centrifugation for 10 min (13000 rpm, 4 °C), 800 μL was loaded into an LC-MS injection vial and waved dry. The cells were re-dissolved in 300 μL of methanol-water (V: V = 1:4) and allowed to stand at - 40 °C for 2 h. After centrifugation for 10 min (13000 rpm, 4 °C), 150 μL of supernatant was aspirated, filtered using a 0.22 μm organic phase pinhole filter, transferred to LC injection vials, and stored at -80 °C. LC-MS analysis was conducted via a LC-MS

system consisting of an ACQUITY UPLC I-Class plus ultra-high performance liquid series QE HF high resolution mass spectrometer. The column was the ACQUITY UPLC HSS T3 (100 × 2.1 mm, 1.8 um). Column temperature: 45 °C; Mobile phase: A-water (containing 0.1% formic acid), B-acetonitrile (containing 0.1% formic acid); Flow rate: 0.35 mL/min; Injection volume: 2 μL; Ion source: ESI. The signal was collected in the positive and negative ion scanning mode.

Supplemental figures

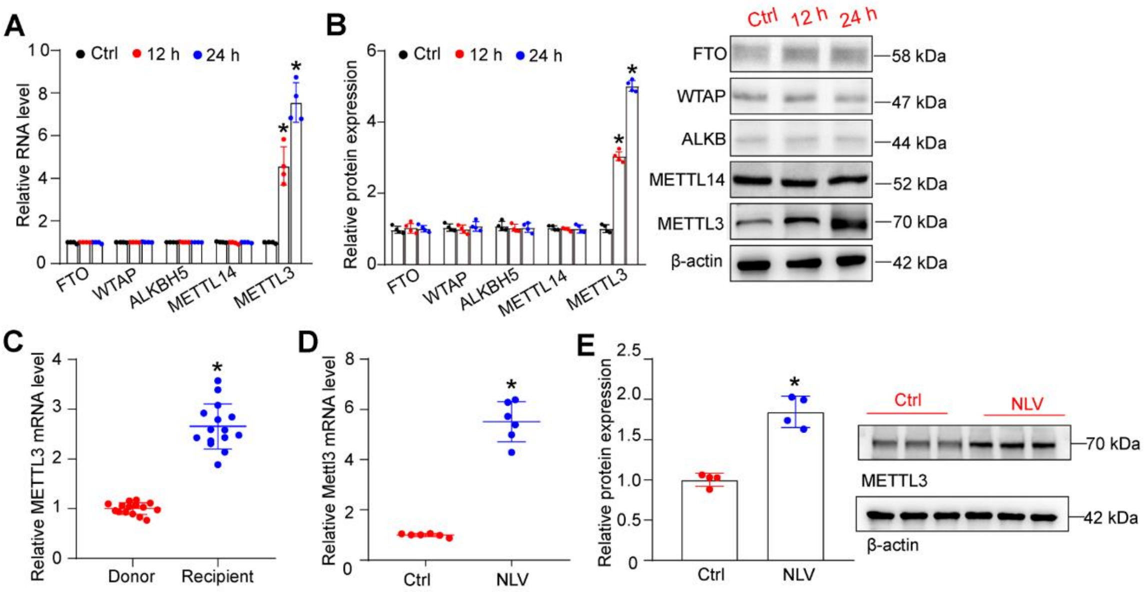

Figure S1: Lymphangiogenesis up-regulates METTL3 expression

(A and B) HLECs were incubated with LPS (1 μg/mL) to mimic inflammatory stress for 12 h or 24 h. The group without LPS treatment was taken as the control (Ctrl) group. qRT-PCR assays (A) and western blots (B) were conducted to detect the levels of METTL3, METTL14, WTAP, FTO, and ALKBH5 (n = 4, One-way ANOVA with Bonferroni test, *$P < 0.05$ versus Ctrl group). (C) qRT-PCR assays were conducted to detect the levels of METTL3 mRNA in the injured corneas and the corresponding donors (n = 15, Mann-Whitney *U* test with Bonferroni test, *$P < 0.05$ versus donor group). (D) qRT-PCR assays were conducted to detect the levels of *Mettl3* mRNA in the sutured corneas and the corresponding controls (n = 6, Mann-Whitney *U* test with Bonferroni test, *$P < 0.05$ versus Ctrl group). (E) Western blots were conducted to detect Mettl3 protein levels in the sutured corneas and the corresponding controls with 3 technical replicates. β-actin was detected as the loading control (n = 4 biological replicates). The representative immunoblots were shown.

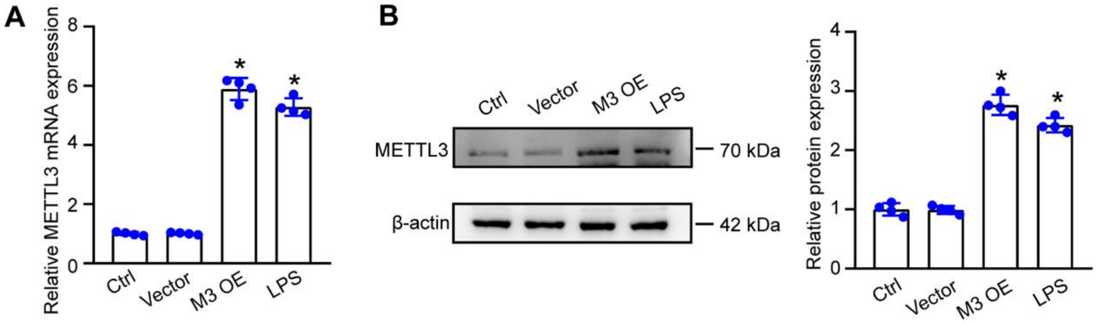

Figure S2: METTL3 overexpression or inflammatory stress up-regulates METTL3 expression in lymphatic endothelial cells

HLECs were transfected with pcDNA 3.1 vector (Vector), pcDNA 3.1-METTL3 (M3 OE), left untreated (Ctrl) for 24 h, or exposed to LPS (1 μg/ml) for 24 h. The levels of METTL3 mRNA were determined by qRT-PCR assays (A, n = 4, one-way ANOVA with Bonferroni test, *$P < 0.05$ vs. Ctrl group). Western blot analysis was performed to assess METTL3 protein levels, with β-actin serving as a loading control (B).

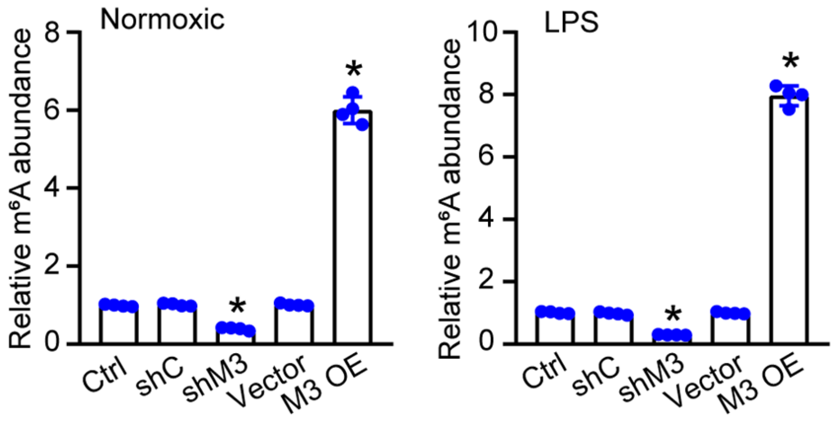

Figure S3: METTL3 intervention affects m^{6}A RNA abundance in lymphatic endothelial cells

HLECs were transfected with METTL3 shRNA (shM3), negative control shRNA (shNC), METTL3 (M3 OE), vector, or left untreated (Ctrl) and then cultured under normal condition and inflammatory condition (LPS exposure) for 24 h. m^{6}A abundance was determined by colorimetric quantification assay (n = 4, one-way ANOVA with Bonferroni test, *$P < 0.05$ versus Ctrl group).

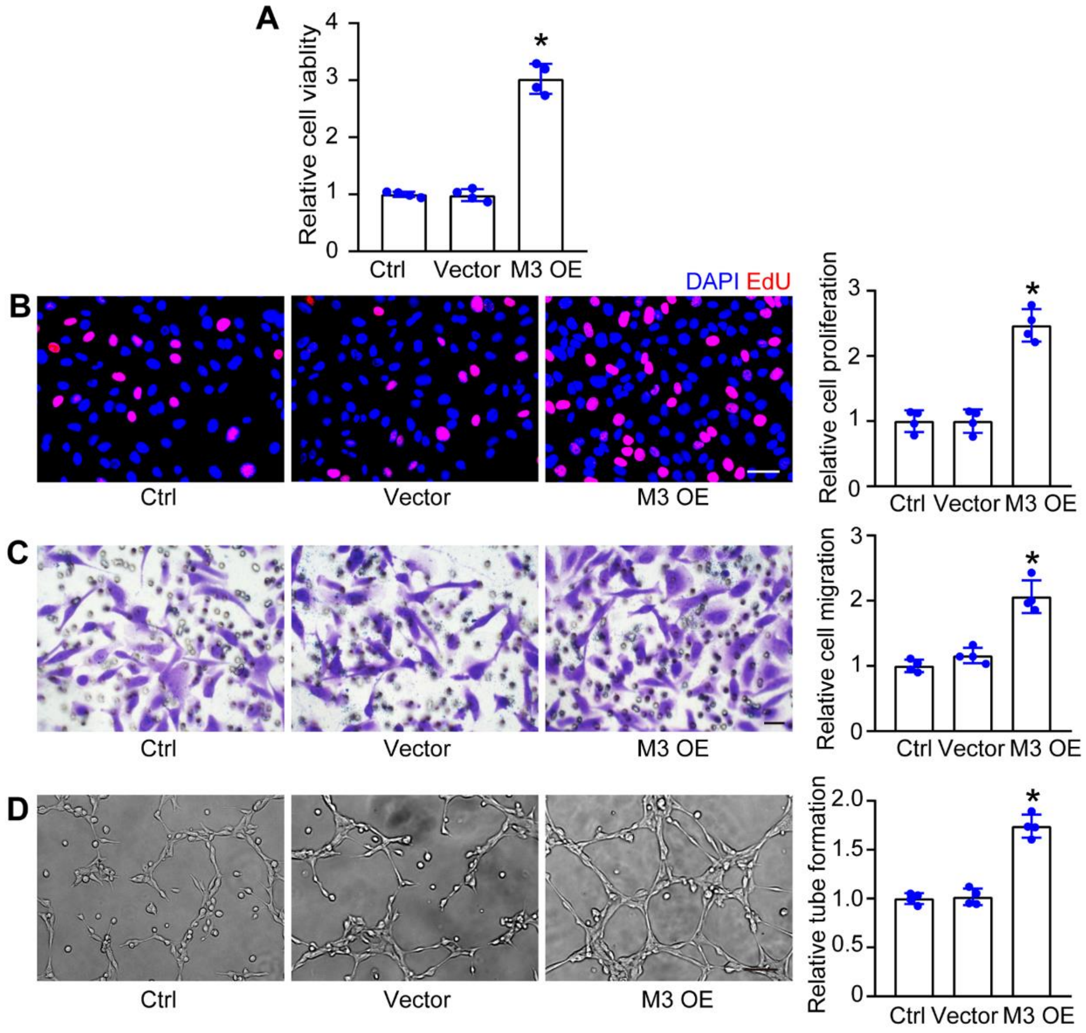

Figure S4: METTL3 overexpression regulates lymphatic endothelial function *in vitro*

HLECs were transfected with pcDNA3.1 (vector), pcDNA3.1-METTL3 (M3 OE), or left untreated (Ctrl) for 24 h. Cell viability was detected by MTT assays (A). EdU staining and quantification analysis was conducted to detect cell proliferation. Scale bar: 20 µm (B). Transwell assay and quantification analysis was conducted to detect cell migration. Scale bar: 20 µm (C). HLECs were seeded on Matrigel matrix and the tube-like structures were observed at 6 h following cell seeding. Average tube length for each field was statistically analyzed. Scale bar: 50 µm (D), n = 4, One-way ANOVA with Bonferroni test, *$P < 0.05$ vs. Ctrl group.

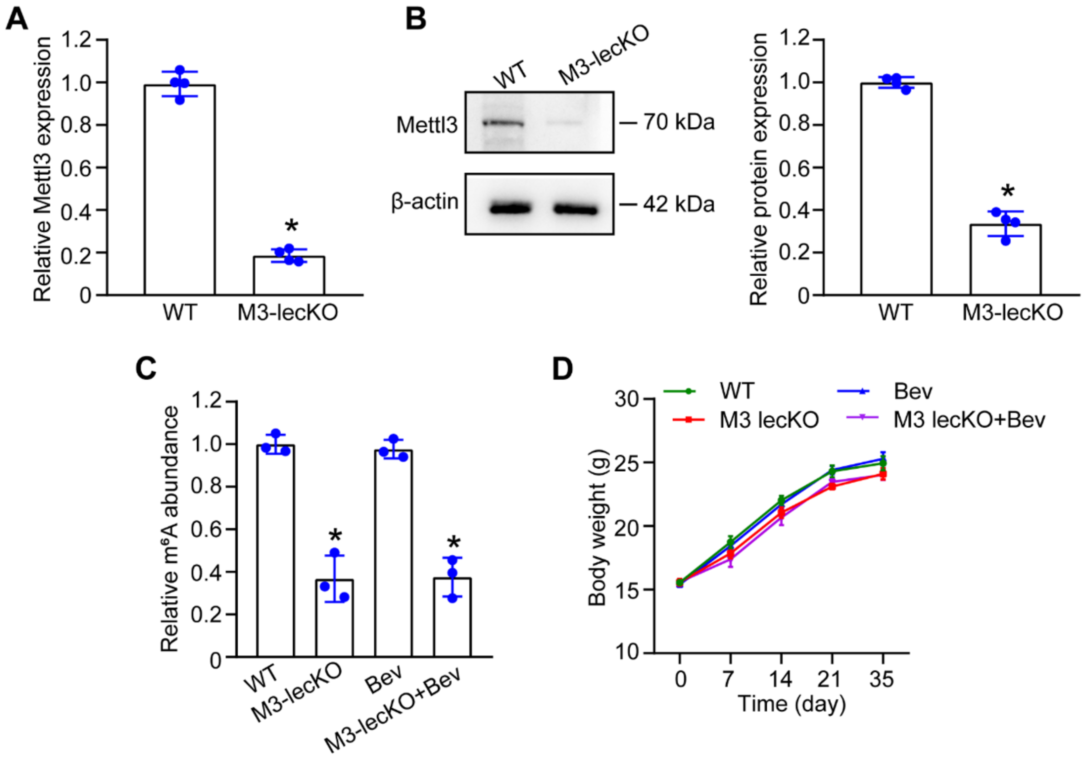

Figure S5: Detection of METTL3 expression, m^{6}A abundance and body weight of Mettl3-lecKO mice.

(A) qRT-PCR assays were conducted to detect Mettl3 mRNA levels in the corneas isolated from wild-type group (n = 4, WT) and Mettl3-lecKO mice (n = 4, *$P < 0.05$ vs. WT group, One-way ANOVA with Bonferroni test). (B) Western blots were conducted to detect the expression of Mettl3 protein. β-actin was detected as the loading control (n = 4). The representative immunoblots were shown. (C) The levels of m^{6}A RNA modification were detected by colorimetric quantification (n = 4, *$P <$ 0.05 vs. WT group, One-way ANOVA with Bonferroni test). (D) The changes of body weights of the mice in intratumoral models (n = 6, One-way ANOVA with Bonferroni test).

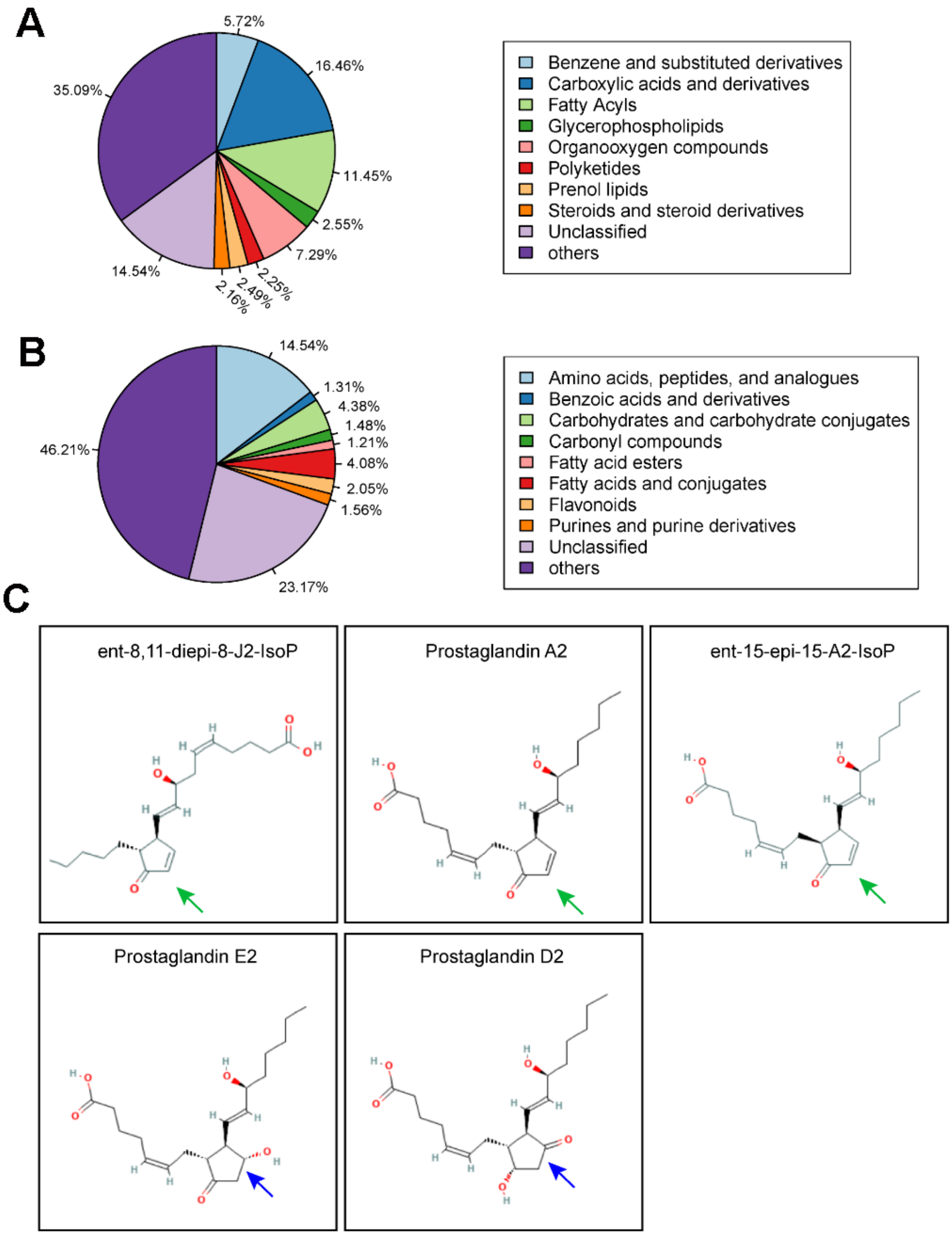

Figure S6: METTL3 knockdown regulates the synthesis of lipids and lipid-like molecules in lymphatic endothelial cells

(A and B) Pie charts show the distribution of differential metabolites by class (A) and sub class (B). (C) The characteristic chemical structures of cyclopentenone prostaglandins and non-cyclopentenone prostaglandins. Green arrow: cyclopentenone ring structure with highly reactive α, β-unsaturated carbonyl group; Blue arrow: saturated six-membered ring.

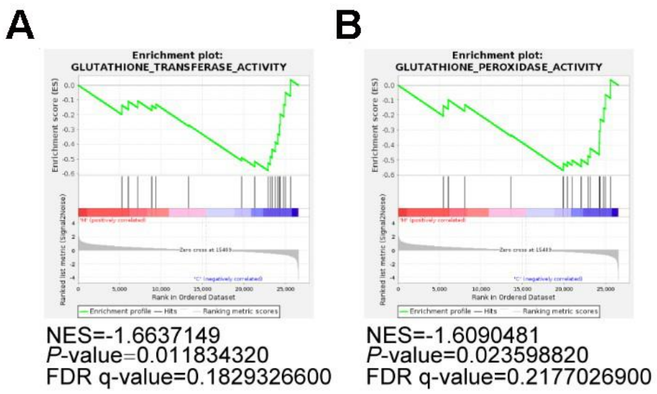

Figure S7: METTL3 knockdown regulates glutathione transferase and oxidase activities in lymphatic endothelial cells

GSEA pathway analysis from RNA-Seq results showed an inverse correlation between the activities of glutathione transferase and oxidase and METTL3 knockdown.

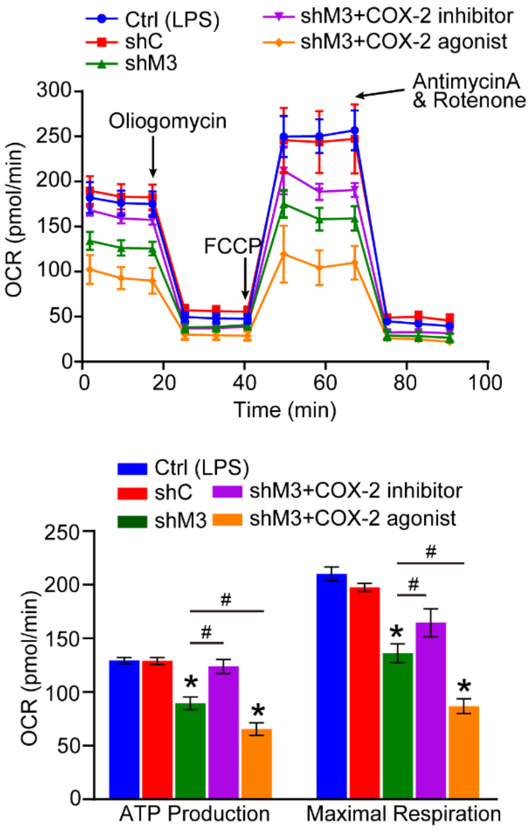

Figure S8: METTL3 knockdown reduces oxygen consumption rate in HLECs upon inflammatory stress

HLECs were treated as shown for 24 h and then exposed to LPS (1 μg/ml) to mimic inflammatory stress for 24 h. ATP production and maximal respiration of HLECs were calculated based on oxygen consumption rate (OCR). $^{*}P < 0.05$ vs. Ctrl (LPS) group; $^{\#}P < 0.05$ between the marked groups; One-way ANOVA with Bonferroni test.

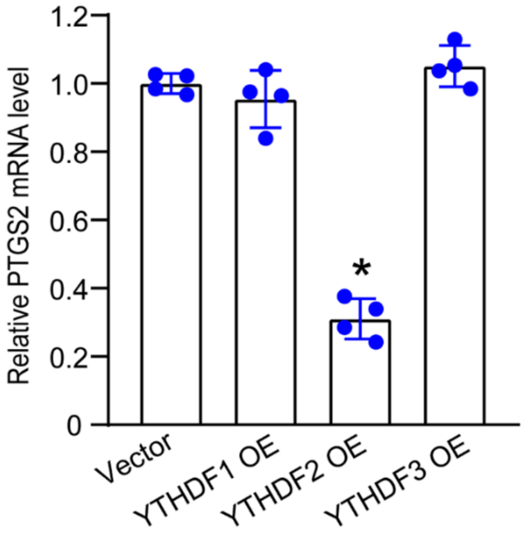

Figure S9: The level of PTGS2 mRNA is reduced response to YTHDF2 overexpression

HLECs were transfected with pcDNA3.1 (vector) or pcDNA3.1-YTHDF1-3 for 24 h. qRT-PCR assays were conducted to detect the levels of PTGS2 mRNA (n = 4, **P* < 0.05 vs. Vector group, One-way ANOVA with Bonferroni test).
